# Sensitivity and specificity of computer vision classification of eyelid photographs for programmatic trachoma assessment

**DOI:** 10.1371/journal.pone.0210463

**Published:** 2019-02-11

**Authors:** Matthew C. Kim, Kazunori Okada, Alexander M. Ryner, Abdou Amza, Zerihun Tadesse, Sun Y. Cotter, Bruce D. Gaynor, Jeremy D. Keenan, Thomas M. Lietman, Travis C. Porco

**Affiliations:** 1 Francis I. Proctor Foundation, University of California San Francisco, San Francisco, CA, United States of America; 2 Department of Computer Science, San Francisco State University, San Francisco, CA, United States of America; 3 Department of Mathematics, San Francisco State University, San Francisco, CA, United States of America; 4 Programme FSS/Université Abdou Moumouni de Niamey, Programme National de Santé Oculaire, Niamey, Niger; 5 Carter Center, Addis Ababa, Ethiopia; 6 Department of Ophthalmology, University of California San Francisco, San Francisco, CA, United States of America; 7 Department of Epidemiology and Biostatistics, University of California San Francisco, San Francisco, CA, United States of America; Centers for Disease Control and Prevention, UNITED STATES

## Abstract

**Background/aims:**

Trachoma programs base treatment decisions on the community prevalence of the clinical signs of trachoma, assessed by direct examination of the conjunctiva. Automated assessment could be more standardized and more cost-effective. We tested the hypothesis that an automated algorithm could classify eyelid photographs better than chance.

**Methods:**

A total of 1,656 field-collected conjunctival images were obtained from clinical trial participants in Niger and Ethiopia. Images were scored for trachomatous inflammation—follicular (TF) and trachomatous inflammation—intense (TI) according to the simplified World Health Organization grading system by expert raters. We developed an automated procedure for image enhancement followed by application of a convolutional neural net classifier for TF and separately for TI. One hundred images were selected for testing TF and TI, and these images were not used for training.

**Results:**

The agreement score for TF and TI tasks for the automated algorithm relative to expert graders was *κ* = 0.44 (95% CI: 0.26 to 0.62, *P* < 0.001) and *κ* = 0.69 (95% CI: 0.55 to 0.84, *P* < 0.001), respectively.

**Discussion:**

For assessing the clinical signs of trachoma, a convolutional neural net performed well above chance when tested against expert consensus. Further improvements in specificity may render this method suitable for field use.

## Introduction

Millions of people are currently blind because of trachoma worldwide, a result of infection by ocular strains of *Chlamydia trachomatis*. [1, 2] This infection is treatable using single-dose azithromycin, and mass administration of azithromycin forms the basis of the World Health Organization’s strategy for trachoma control. [3] Stakeholders base decisions on starting programs, stopping mass treatment, and declaring control on the clinical signs of trachoma. Yet studies show a great deal of variance between graders, or even the same grader over time. If any concerns later arise, field results are not auditable. Photographic grading appears to be as accurate as clinical grading, and could overcome other limitations. [4]

Is it possible for an automated algorithm to clinically grade active trachoma from photographs collected in the field? We note that automated image processing is becoming useful in many medical imaging applications. [5–7] Our application differs from most in that we use images collected under field conditions (under differing lighting conditions and camera angles and distances), and in that we are conducting classifications of a subclinical condition with an ultimate goal of guiding, not individual treatment, but community-wide mass administration of azithromycin for a public health control campaign. Automated scoring would avoid human grader drift over time. [8, 9] It would also permit standardization of methods between regions and countries, and could allow a higher volume of images to be scored at lower cost. Neural networks have long been useful for diagnostic tests in medicine, and in ophthalmological applications in particular. [10–12] Here, we test the hypothesis that a convolutional neural network [13] can classify trachoma photographs substantially better than chance.

## Materials and methods

### Data

Images used in this prospective study were obtained from two clinical trials: the Niger arm of the Partnership for the Rapid Elimination of Trachoma trial (PRET, clinicaltrials.gov accession number NCT00792922), and the Trachoma Amelioration in Northern Amhara trial (TANA, clinicaltrials.gov accession number NCT01202331). These trials included a total of 85550 participants, with details published elsewhere. [14, 15] Verbal consent was obtained for study subjects, and ethical approval was obtained from the University of California, San Francisco, the Niger Ministry of Health, and the Ethiopian Ministry of Science and Technology.

**Fig 1:**
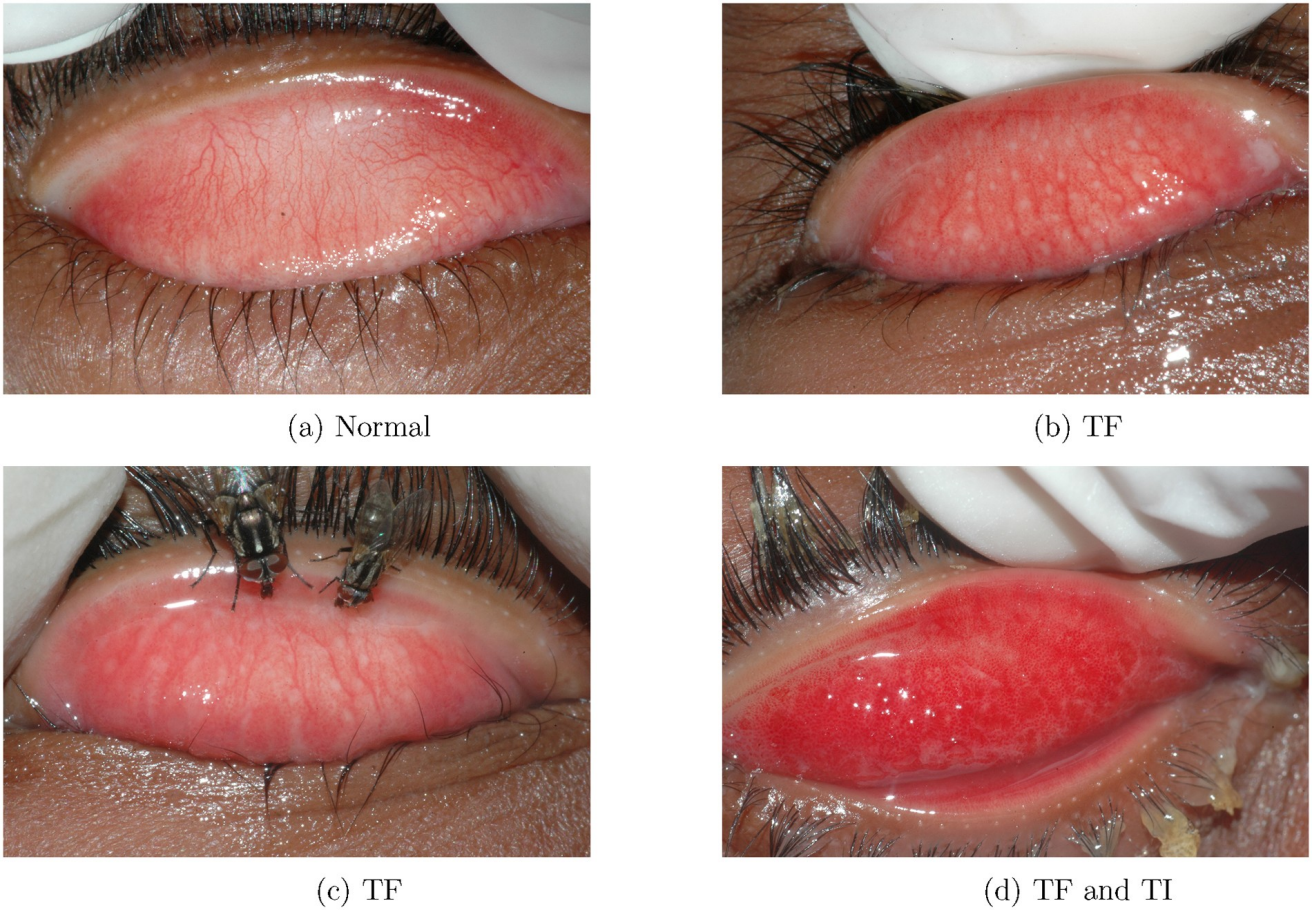
Trachoma classification of selected field collected images, according to the WHO simplified system. TF: trachomatous inflammation—follicular; TI: trachomatous inflammation—intense [16].

Images were taken by community health workers who were trained in field trachoma evaluation, and who were implementing the specimen collection for each trial. For each study participant, the right upper eyelid was everted and the underlying tarsal conjunctiva photographed with a single-lens reflex (SLR) camera equipped with a 105/2.8*f* macro lens using a standardized protocol (aperture priority, aperture *f*/40, ISO 400, native flash engaged, automatic white balance, at least 2 high-quality photographs taken). Images were saved in JPG format. A panel of three experts applied the WHO simplified system [16] to randomly selected images. The graders classified each image for the presence or absence of TF and the presence or absence of TI, independently. No qualitative evaluation of TF or TI intensity was conducted (see Fig 1). The three human experts graded images independently, each masked to the grades of the other two. A labeled data set of 1,656 digital images was obtained, considering the human consensus as the gold standard (Table 1). No missing or indeterminate grades were allowed. These images were used in assessment of field grading for the clinical trials, and were the total set of available images. Each image in our dataset exhibits an everted eyelid that is approximately centered and parallel to the edge of the photograph.

**Table 1:** Distribution of clinical categories in our dataset.

Label	Number	% of total	Sub categories		
Neither TF nor TI	843	51.0%			
Infected	813	49.0%	TF	527	32.0%
			TI	272	16.3%
			TI and TF	162	9.7%
			Scarring	176	10.6%

For both TF and TI cases, we randomly sampled 50 images from the TF or TI labeled set and another 50 images from the normal set to obtain a hold-out validation set. These images were not used to train classifiers, but were used only to produce the final performance scores.

For TF classification, we utilized 477 images scored as TF and 793 normal images; for TI classification, we utilized 222 images scored as TI alone and the same 793 normal images. These constituted a random sample of images that had been prepared for trial evaluation. We estimated that inclusion of 230 images would achieve an estimated standard error of 0.05 in Cohen’s kappa, assuming *κ* = 0.8 and that 20% of the images were classified TF.

### Machine classification

#### Image preprocessing

**Fig 2:**
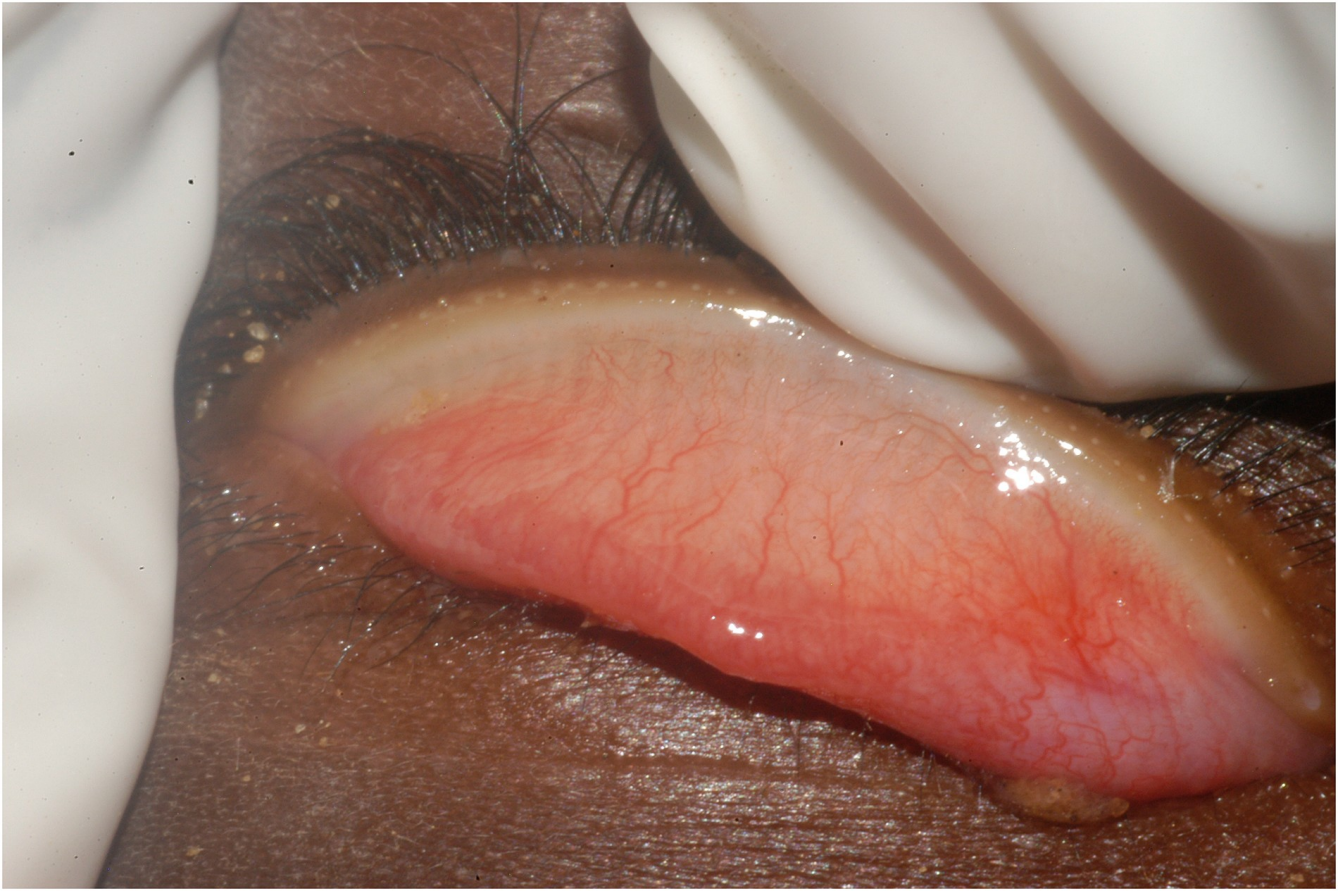
Sample image where eyelid is neither centered nor horizontally aligned.

**Fig 3:**
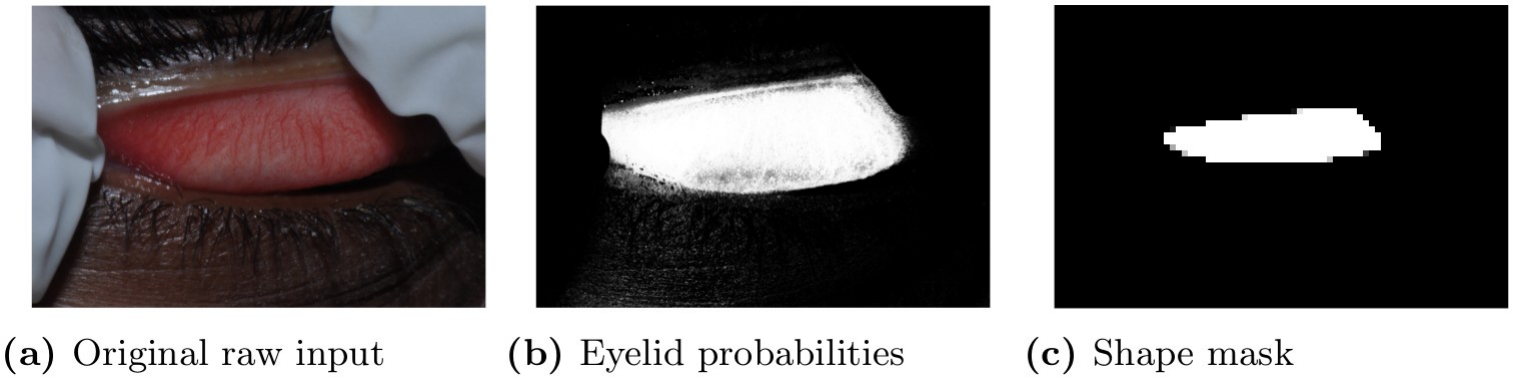
An illustrative example for various eyelid images in our procedural pipeline.

Automated preprocessing was necessary, since the eyelid may have been off center or misaligned with the edge (Fig 2). The eyelid in each image was approximately centered and parallel to the main axis of the photograph as shown in Fig 3(a). The region of interest was automatically extracted without these assumptions using a four step procedure consisting of image resizing, application of a pixel-level classifier, a corrective rotation step, and finally a crop to yield a standard size region of interest. Note that pixel classifiers have proven useful in other applications [17].

Resizing. The original raw images are in color JPEG format, which vary in dimension from 4288 × 2848 to 3008 × 2000 pixels. The first step in preprocessing was resize the images to 1024 × 680 preserving the 3:2 ratio of the digital cameras using the image resizing function in the OpenCV package [18] with linear interpolation. Our eyelid rectification procedure then transforms these preprocessed color images into cropped grayscale images of size 128 × 128 containing an eyelid in a standard orientation and location. This procedure consists of the three successive steps: 1) a pixel-level transformation, 2) a corrective rotation, and 3) a ROI crop selection. We explain these steps in some detail below.

Classification of pixels. We used a pixel-level classifier as part of the image preprocessing; a different classifier is used for classifying the entire image into trachoma-related categories. For the first pixel-level transformation step, we build a binary classifier that maps a pixel color in RBG values into the probability of the pixel being on an eyelid or not. This classifier is then successively applied to each pixel of the 1024 × 680 preprocessed image, yielding a probabilistic image of the same size, whose pixel value represents the estimated probability that certain pixel belongs to an eyelid (See Fig 3(b) for an example).

**Fig 4:**
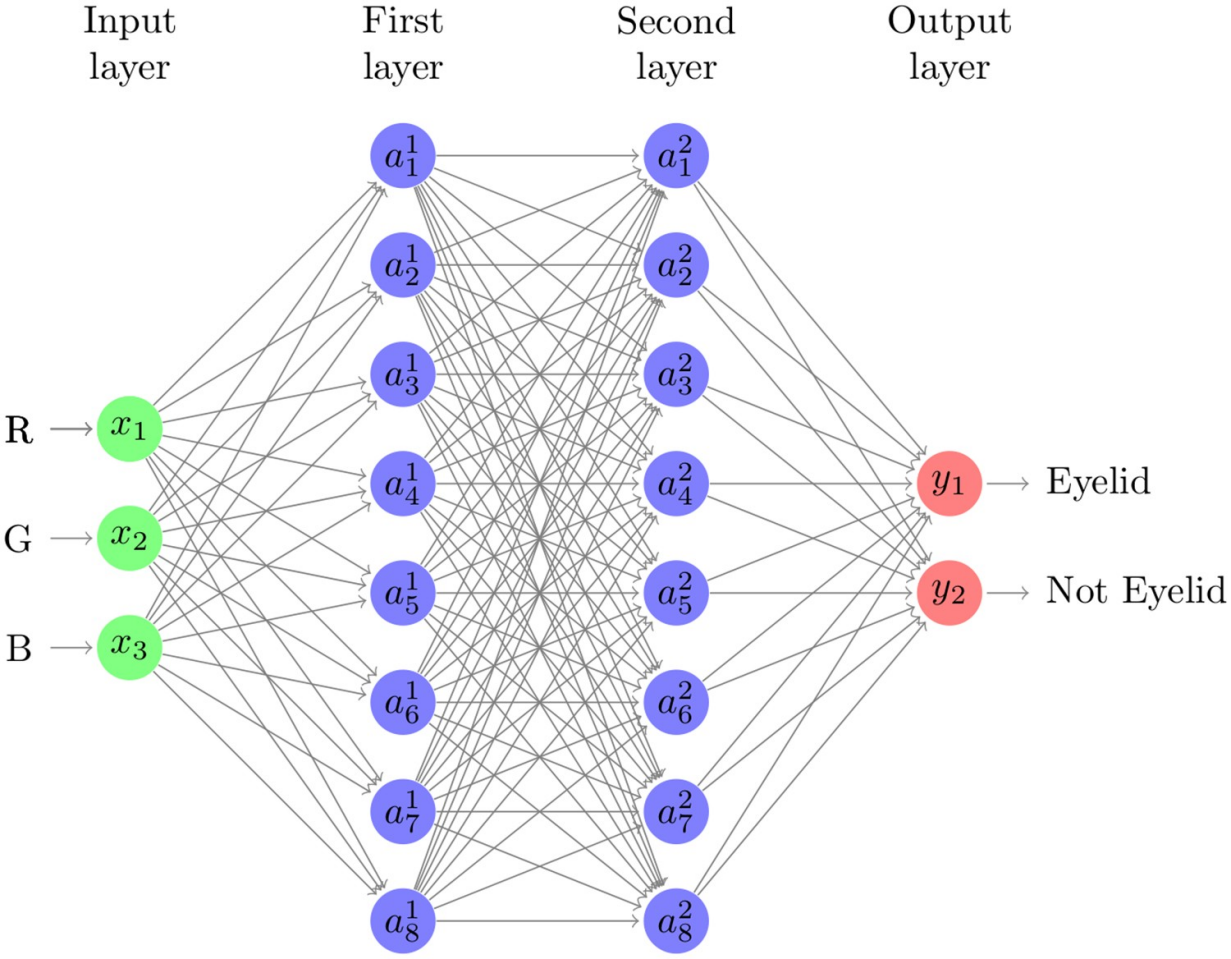
Network architecture of multilayer perceptron-based pixel-level classifier.

We design this classifier with a multilayer perceptron [19] with two fully-connected hidden layers. Architectural overview of this network is shown in Fig 4. The input layer to the multilayer perceptron consists of three neurons (*x*_1_, *x*_2_, *x*_3_) corresponding to a pixel’s RGB values between 0 and 255. The first and the second hidden layer includes 8 neurons $\left(a_{1,‥,8}^{1,2}\right)$. The final output layer consists of two neurons (*y*_1_, *y*_2_), representing two possible states: whether a pixel is on eyelid or not. For both the input and hidden layers the rectified linear unit (ReLu) defined as *f*(*x*) ≔ max(*x*, 0) is used as its non-linear activation function. The softmax function is applied to the output neurons in order to generate a two dimensional stochastic vector estimating the probability distribution of the pixel belonging to an eyelid.

We train this classifier by backpropagation [20] with the stochastic gradient descent and the categorical cross-entropy as its loss function. [21] The training set, consisting of 32 million positive (i.e., eyelid) pixels and 41 million negative (i.e., non-eyelid) pixels, was prepared by hand-segmenting eyelids in 40 images randomly sampled from our training set for positives and by collecting 28 non-eyelid crops for negatives, including various types of objects such as skin, fingers, eyelashes, and insects. Our trained classifier yielded 96.7% accuracy when tested with our hold-out validation data sets for validation.

Corrective rotation. For the second corrective rotation step, we first estimate an eyelid’s center and its binary shape mask from the result of the first step, then perform discrete Gabor transform on the shape mask in order to estimate the tilt-angle between the major axis of the detected eyelid and the horizontal image axis. The image is then rotated to correct this tilt, resulting in automatic alignment of the eyelid’s orientation.

Given the image of estimated probabilities from the first step, we first smooth this probabilistic field by 3 × 3 median filtering. Then we estimate the location of the everted eyelid’s center by computing the centroid of the probabilistic field. We also derive the binary eyelid shape mask (see Fig 3(c) for an example) by thresholding the probability value *p* at each pixel: eyelid (1) if *p* > *TH* and non-eyelid (0) otherwise. We use TH = 0.6 that was empirically chosen. The resulting binary field is then smoothed by morphological closing. [22] We estimate the tilt-angle of the eyelid’s major axis by 1) convolving the shape mask at the center location with a bank of 18 orientation-selective discrete Gabor filters [22, 23] designed in a range between $-\frac{\pi }{4}$ and $\frac{\pi }{4}$ with an interval of $\frac{\pi }{36}$, and 2) selecting the filter that resulted in the maximum response. The angle associated with the selected maximal filter is used as our tilt-angle estimate. The original image is then rotated by the negative of this angle to align the eyelid horizontally.

**Fig 5:**
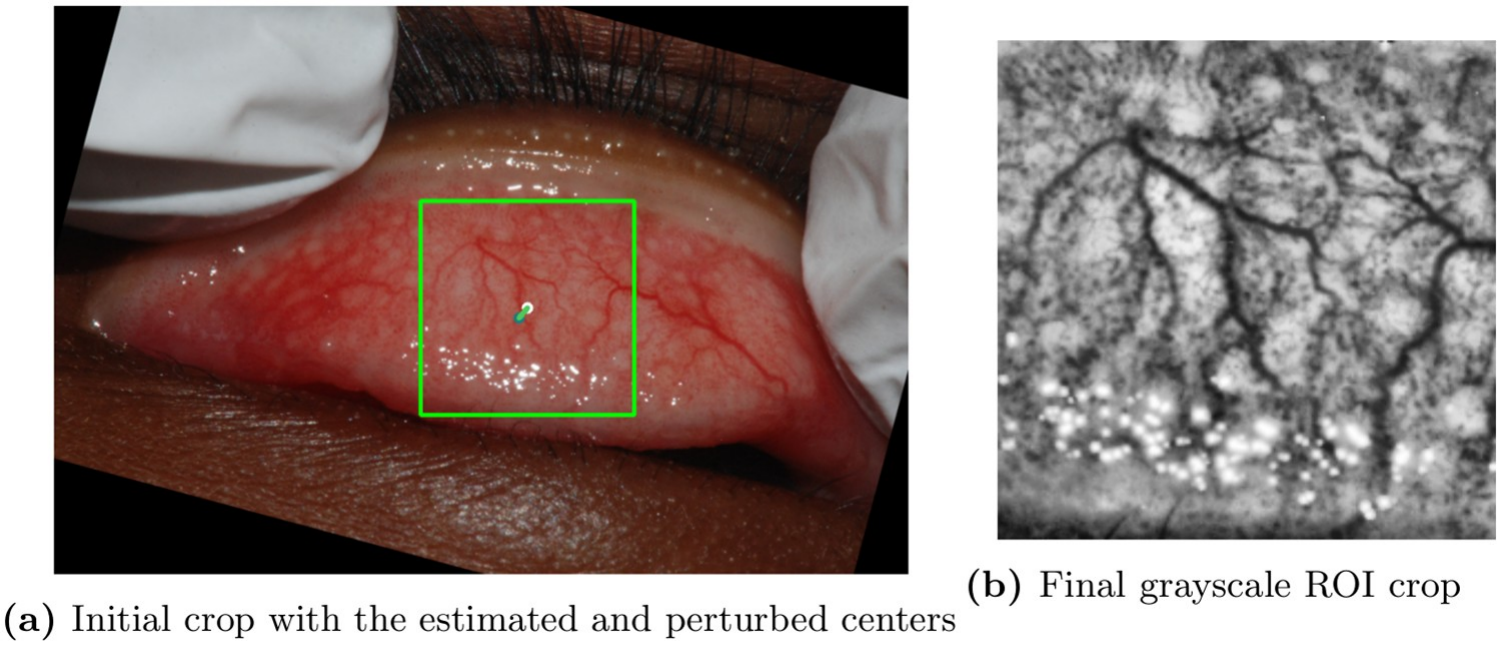
ROI crop selection procedure. (a) 256 × 256 crop on the rotated image. Estimated (white) and randomly perturbed eyelid centers (green) are shown. (b) Resulting 128 × 128 grayscale ROI.

Crop. For the final ROI crop selection step, we first extract, from the preprocessed and rotated image, a 256 × 256 crop centered at the eyelid center estimated in the previous step. We did not estimate the eyelid size for each image since our data set came with relatively similar size of eyelids across images. The window size was empirically chosen to encompass the extent of eyelids across images. The crop is then resized to 128 × 128 and converted to gray scale. We then applied contrast limiting adaptive histogram normalization [24] in order to enhance and standardize grayscale contrasts. Fig 5 shows an illustrative example for this final step.

#### Trachoma Classification

This section describes our classification model that takes the 128 × 128 region of interest from the previous eyelid rectification process as an input, and outputs the binary classification of whether an eye depicted in the input image exhibits the signs TF or TI. We designed our model with a convolutional neural network [13]. Our network consists of three stage convolutional layers followed by fully connected layers with two hidden layers. Note that the representational power of a convolutional neural network is not compromised by the use of relatively small (3 × 3) filters, since by stacking several convolutional layers, a much larger effective field is realized. [13] The Keras platform with a Theano backend was used in implementation (https://keras.io/, http://deeplearning.net/software/theano).

**Fig 6:**
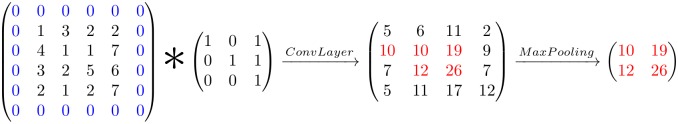
Convolutional layer with zero-padding and a 3 × 3 filter followed by max pooling with a 2 × 2 block.

Table 2 summarizes our convolutional neural network architecture. We use convolutional filters of size 3 × 3. Each stage of the convolutional layers is augmented with a max-pooling layer with 2 × 2 size blocks, halving the size of the input after each stage. The border of input image is zero-padded before convolution of each layer. Fig 6 illustrates computational procedures in the convolutinoal layer for a schematized simple example. Two fully-connected hidden-layers of our network include 512 neurons. The final output layer consists of two positive/negative neurons whose value indicates the probability that the target image manifests TF or TI, respectively. The rectified linear unit (ReLu), defined above, is used as our activation function of each layer. The softmax function was applied to the final output layer to produce a probabilistic classification. Final binary classification is then given by thresholding the probability with *TH* = 0.5.

**Table 2:** Architecture of our convolutional neural network classification model. *K* denotes the number of filters in the first stage of the convolutional layers.

Layer	Size
Convolution 1.1	3 × 3 × *K*
Convolution 1.2	3 × 3 × *K*
Max Pooling 1	2 × 2
Convolution 2.1	3 × 3 × 2*K*
Convolution 2.2	3 × 3 × 2*K*
Max Pooling 2	2 × 2
Convolution 3.1	3 × 3 × 4*K*
Convolution 3.2	3 × 3 × 4*K*
Convolution 3.3	3 × 3 × 4*K*
Max Pooling 3	2 × 2
Fully-Connected Hidden 1	512
Fully-Connected Hidden 2	512
Fully-Connected Output	2

Training. For training, we used the binary cross-entropy loss function [21] with respect to the gold standard labels made by our expert panels. We then trained our classification model using two learning strategies of 1) a standard stochastic gradient descent algorithm [25] and 2) AdaDelta [26] with an adaptive learning rate. Furthermore, we tested our model with the varying number of convolutional filters *K* set to 8, 16, 32, or 64. The best strategy with the maximum performance was determined by using a second hold-out test set which was prepared by randomly selecting 10% of the training set, described in Data section, to be used for computing performance statistics of a model trained with the remaining 90% in order to minimize over-fitting.

To further reduce the possibility of overfitting, we incorporated the following strategies during our model training. We used batch normalization [27] after each set of convolutional layers at training time, which normalizes batches of training images in between layers so that each pixel has a standard normal distribution over all the images in the batch. This prevents variations in the distribution of training data in deeper networks, which can slow training by forcing the later weights to accommodate a larger domain. We also employed random dropout [28] of 25% (i.e. 25% of weights are randomly chosen to be turned off, forcing the remaining weights to generalize faster) after each stage of convolutional layers. Training for the fully-connected layers were also subject to *L*2 regularization with a quadratic complexity penalty. We utilized the same strategies for building models for both TF and TI classification tasks.

We used an additional strategy to reduce overfitting. Specifically, we augmented our cropped region of interests as follows (see Fig 5(b)) As described above, we used an automated procedure which yields a single region of interest crop centered at the estimated eyelid center location. As shown in Fig 5(a), we modified this procedure to introduce randomly generated noise to the centroid location of each eyelid. Repeating the procedure with this perturbed eyelid center yields a new cropped region of interest which exhibits a slightly translated view from the original crop. In order to effectively increase the size of the training set, we incorporated this random data perturbation between each successive epoch (i.e., iteration) during our model training, providing, in essence, a virtually unlimited stream of new training images.

## Results

We trained eight convolutional neural network models by varying the two learning strategies and four *K* values for each of TF and TI classification tasks. Using the hold-out set, we ranked eight models in terms of the kappa statistic for each of the two TF and TI tasks.

The model that produced the best training-time scores on the TF task was trained using AdaDelta and used *K* = 64 filters in its initial convolutional layer. The best performing model for the TI task was training using stochastic gradient descent and used *K* = 32 filters. We compared this best performing model with an ensemble classifier that averages the output probabilities estimated by the three top models for each task. Finally, for validating the best and the ensemble classifiers for the TF and TI tasks, we used the first hold-out validation set of 100 cases for each task and computed four standard performance statistic scores: sensitivity, specificity, accuracy, and Cohen’s kappa (*κ* [29]). Table 3 summarizes the results.

**Table 3:** Validation scores on trained convolutional neural network models for TF and TI classification tasks.

Class	Measure	Best Model	Ensemble of Top-3
TF	*κ*	0.40	0.44
	Sensitivity	0.92	0.86
	Specificity	0.48	0.58
	Accuracy	0.70	0.72
TI	*κ*	0.69	0.69
	Sensitivity	0.98	0.96
	Specificity	0.72	0.74
	Accuracy	0.85	0.85

We observe in the results that the ensemble classifiers yield better performance than the separate classifiers, measured by the kappa, specificity, and accuracy scores for both TF and TI tasks. However the ensemble decreases the sensitivity (e.g., recall) score. The results also indicate that scores for the TI task are higher than those for the TF task for all four measures and for both best and ensemble classifiers. The agreements for TF and TI tasks by our ensemble models were *κ* = 0.44 (95% CI: 0.26 to 0.62, *P* < 0.001) and *κ* = 0.69 (95% CI: 0.55 to 0.84, *P* < 0.001), indicating results far better than chance.

## Discussion

We found that machine classification of field collected eyelid images can yield automated trachoma classifications with performance far better than expected from chance alone. Confining our attention to studies with digital images, we note that human grading of conjunctival photographs using the same protocol resulted in a Cohen’s kappa of 0.55 in one study, and direct conjunctival examination in the field has shown agreement in the range of 0.57 to 0.73. [4, 8]

Trachoma grades TF and TI are defined by the presence of specific features on eyelids (seen in the field or through photographs), as assessed by human experts. No other gold standard for the clinical grade is available. The agreements for TF and TI tasks by our ensemble models were lower and higher (respectively) than this baseline reported [4]. We note that in the Global Trachoma Mapping Project [30], the protocol required that the agreement for TF between a master grader and candidate grader trainer should be at least 0.8 for certification of the candidate grader.

Our experimental results also suggest that TI classification may be easier than TF classification. The best kappa was 0.44 and 0.69 for the TF and TI tasks both by the ensemble classifiers, respectively. The network trained for the TI task outperformed that for the TF tasks for all scores. Although not currently used programmatically, the TI classification appears to be much more specific than TF, and may be more correlated with actual chlamydial infection. [9] Overall, these reasonably high validation scores are promising toward further improving the proposed methodologies to our goal of deploying such automated grading software for the actual field studies.

We note certain limitations. We did not explore various representation of color information beyond the RGB space, nor classification models other than the chosen multilayer perceptron for the eyelid detection. We have no information on generalizability beyond the two countries examined, to archival images, or to images collected with smartphones. Our algorithm was designed for assessment of images as part of a trachoma control campaign, not for individual-level assessment. Such a classifier could enable the assessment of community and district level TF prevalence, as needed to guide intervention efforts during the WHO trachoma elimination campaign. [3] Thus, we have not trained the system to evaluate other features, since active trachoma is usually a subclinical condition which poses no immediate threat to vision. The proposed method, in principle, could be extended to more detailed trachoma classifications. [31, 32]

## Conclusion

Although grading of field trachoma images can be challenging due to less standardization of lighting and distance than in other computer vision exercises, we showed that computer vision methods are capable of classifying field collected trachoma images better than chance. Use of newer deep learning algorithms, together with larger corpuses of labeled trachoma images which are becoming available, is expected to yield substantial improvements in specificity of classification. This may thus permit computer vision techniques to now play a practical role in preserving human vision in some of the world’s poorest communities.
